# Voluntary running partially prevents photoreceptor cell death in retinitis pigmentosa

**DOI:** 10.3389/fnins.2025.1563607

**Published:** 2025-04-25

**Authors:** Stephen K. Agadagba, Ying Liang, Kristine N. Dalton, Benjamin Thompson, Suk-Yu Yau

**Affiliations:** ^1^Centre for Eye and Vision Research Limited, Hong Kong Science Park, Hong Kong, China; ^2^School of Optometry and Vision Science, University of Waterloo, Waterloo, ON, Canada; ^3^Department of Rehabilitation Sciences, The Hong Kong Polytechnic University, Hong Kong, China

**Keywords:** retinitis pigmentosa, photoreceptor degeneration, adiponectin signaling, voluntary exercise, mitochondrial biogenesis

## Abstract

Retinitis pigmentosa (RP) is a progressive retinal degenerative disorder characterized by photoreceptor cell death, leading to vision loss. Current treatments are limited, and there is a need for non-invasive interventions. This study evaluates the neuroprotective effects of voluntary exercise in an RP mouse model and explores the role of the adiponectin signaling pathway in mediating these effects. Pregnant *Pde*6*b*^*rd*10^ (rd10) mice, a transgenic model of RP, and wild-type C57BL/6J mice were divided into sedentary or voluntary running groups (*n = 4* per group). Offspring were analyzed at 6 weeks for photoreceptor nuclei counts, outer segment lengths, serum and retinal adiponectin levels, and expression of AMPK and PGC-1α proteins using immunohistochemistry, ELISA, and Western blotting. Voluntary exercise significantly preserved photoreceptor nuclei (97 ± 16 vs. 32 ± 5 in sedentary rd10 mice) and outer segment lengths for rods (13.1 ± 1.2 μ vs. 1.1 ± 0.6 μ) and cones (7 ± 0.9 μ vs. 0.2 ± 0.1 μm) compared to sedentary rd10 mice. Serum adiponectin levels increased significantly in exercised rd10 mice (*p* < 0.05), while retinal adiponectin levels were elevated in both sedentary and exercised rd10 mice relative to wild-type controls (*p* < 0.005). No significant changes in AMPK (*p* = 0.724) and PGC-1α (*p* = 0.794) protein levels were observed between exercised and sedentary rd10 mice. These findings suggest that voluntary exercise enhances photoreceptor survival in RP by increasing serum adiponectin levels, potentially contributing to neuroprotection. Elevated retinal adiponectin appears linked to RP pathology rather than exercise-induced changes. This study highlights the therapeutic potential of exercise in RP and identifies adiponectin as a promising target for further investigation into neuroprotective mechanisms and treatments.

## 1 Introduction

Retinitis pigmentosa (RP) is a genetically heterogeneous retinal degenerative disorder characterized by the progressive loss of photoreceptors and retinal pigment epithelial cells, ultimately leading to blindness. This condition affects both eyes and can vary significantly in its progression and severity among individuals (Liu W. et al., 2022; Li et al., 2024). The primary symptoms include night blindness, followed by a gradual loss of peripheral vision, and eventually central vision impairment (Chivers et al., 2021). Photoreceptors, particularly rods, have high energy demands due to their continuous renewal of outer segments and the process of phototransduction. RP drastically attenuates photoreceptor function, which is heavily dependent on proper energy metabolism (Hurley, 2021; Pan et al., 2021; Nolan et al., 2022). The metabolic coupling between photoreceptors and the retinal pigment epithelium (RPE) is crucial for maintaining retinal health. In RP, this coupling is disrupted, leading to glycolytic failure and reduced glucose metabolism in photoreceptors (Wang et al., 2019; Nolan et al., 2022). Current treatments for RP focus on slowing disease progression and managing symptoms, as no cure exists. Gene therapy using techniques like CRISPR/Cas9 and optogenetics are being explored to correct genetic defects and restore some visual function (Kantor et al., 2020; Liu W. et al., 2022). Although stem cell therapy shows promise in early-phase clinical trials, it has not yet been approved for clinical use due to significant barriers such as high cost and technical complexity (Alcalde et al., 2022; Chen et al., 2023). There is a pressing need to develop broadly applicable and effective treatments for RP.

Extensive research highlights the significant benefits of regular physical exercise on neurological health, including retinal health. Physical exercise is known to improve various aspects of neurological function, such as cognitive performance, memory, attention, and executive functions across all age groups (Liu-Ambrose et al., 2010; Nanda et al., 2013; Hussey et al., 2020; Lissek et al., 2024). It promotes the growth and survival of brain cells, increases neuroplasticity, and supports adult hippocampal neurogenesis, which is crucial for learning and memory (Yau et al., 2014; Yau et al., 2015). Furthermore, regular physical exercise is linked to a reduced risk of neurodegenerative diseases, including dementia, Alzheimer’s disease, and Parkinson’s disease (Bacanoiu et al., 2023; Santiago and Potashkin, 2023). Specifically, in the context of retinal health, physical exercise has been shown to have neuroprotective effects, potentially delaying the progression of degenerative conditions like RP. In addition to physical exercise, other non-invasive strategies have been explored for retinal neuroprotection, including dietary interventions and pharmacological approaches. For example, supplementation with omega-3 fatty acids and vitamin A has been investigated for its ability to slow photoreceptor degeneration, with mixed clinical outcomes (Berson et al., 2004; Liu Y. et al., 2022). Neuroprotective agents such as ciliary neurotrophic factor (CNTF) and N-acetylcysteine (NAC) have also been studied for their potential to enhance photoreceptor survival and mitigate oxidative stress (Faktorovich et al., 1990; Lee et al., 2011). Compared to these approaches, physical exercise offers a low-cost, broadly accessible intervention with systemic benefits that extend beyond the retina. Unlike pharmacological or dietary interventions, which rely on exogenous compounds, exercise modulates multiple physiological pathways, including inflammation, metabolism, and neurotrophic support, that may contribute to retinal resilience. However, while studies have demonstrated that voluntary exercise can delay photoreceptor degeneration, the precise mechanisms underlying these effects remain incompletely understood. The evidence supporting physical exercise as a neuroprotective intervention is robust (Agadagba et al., 2024), with preclinical studies in rodent models demonstrating that physical exercise elevates neurotrophic factors such as brain-derived neurotrophic factor (BDNF), which enhance neurotransmission and neurogenesis (Boehme et al., 2011; Venezia et al., 2017). These neuroprotective effects may extend to retinal health, as physical exercise has been found to influence metabolic and cellular pathways critical for photoreceptor survival and function.

The specific effects of physical exercise on retinal health, particularly in the context of RP, are an emerging area of interest. Rodent models of RP, such as the PDE6Brd10 (rd10) mouse model, have provided valuable insights into the potential therapeutic benefits of physical exercise. Research has shown that voluntary exercise can lead to significant improvements in photoreceptor survival and function in these models. For instance, studies have demonstrated that voluntary exercise preserves visual function and reduces the inflammatory response in an adult mouse model of autosomal dominant retinitis pigmentosa (Bales et al., 2024). These findings suggest that physical exercise may help mitigate the degenerative effects of RP by enhancing cellular resilience and metabolic function within the retina. Additionally, physical exercise has been associated with increased levels of neurotrophic and metabolic factors, which may contribute to its protective effects on retinal cells (Yau et al., 2014; Pan et al., 2021). The mechanisms underlying these benefits are thought to involve the upregulation of signaling pathways related to mitochondrial biogenesis and energy metabolism, which are essential for maintaining photoreceptor health and function.

Despite the promising findings on the benefits of physical exercise for retinal health, several aspects remain unknown. One key question is whether the adiponectin signaling pathway plays a crucial role in mediating the neuroprotective effects of physical exercise in the context of RP. Adiponectin, a hormone primarily secreted by adipose tissues was selected as a candidate pathway based on its established roles in neuroprotection, inflammation regulation, and energy metabolism. Adiponectin and its receptors (AdipoR1 and AdipoR2) are expressed in the retina, where they contribute to mitochondrial function, lipid metabolism, and oxidative stress regulation (Fu et al., 2016; Choubey and Bora, 2023). Previous studies have demonstrated that loss of AdipoR1 leads to progressive photoreceptor degeneration, highlighting the importance of adiponectin signaling in retinal homeostasis (Rice et al., 2015). Furthermore, systemic adiponectin has been implicated in protecting retinal neurons from degeneration in models of diabetic retinopathy and retinal ischemia (Shukal et al., 2022). Given these findings, we hypothesized that adiponectin may contribute to exercise-induced neuroprotection in RP by activating pathways associated with photoreceptor survival. Moreover, adiponectin plays a critical role in metabolic regulation through its signaling pathways, particularly the AMP-activated protein kinase (AMPK) and peroxisome proliferator-activated receptor-gamma coactivator-1-alpha (PGC-1α) pathways (Iwabu et al., 2019; Choi et al., 2020). These pathways are known to enhance glucose uptake, fatty acid oxidation, and mitochondrial biogenesis, which are vital for cellular energy balance and metabolic health. In the context of neuroprotection, central adiponectin signaling has been shown to support neuronal plasticity and metabolic health (Arquier et al., 2021). Given these established roles of adiponectin in metabolic regulation and neuroprotection, we hypothesize that voluntary exercise may delay photoreceptor degeneration in RP by increasing adiponectin levels in the retina, thereby activating the AMPK-PGC-1α signaling pathway. This study aims to elucidate the potential neuroprotective effects of physical exercise in a mouse model of RP and to determine the role of the adiponectin signaling pathway in mediating these effects. By bridging the gap between exercise-induced metabolic benefits and their potential therapeutic applications in neurodegenerative diseases like RP, this research seeks to offer new insights into the molecular mechanisms underlying exercise-mediated neuroprotection and suggest novel therapeutic strategies for mitigating photoreceptor loss in RP patients.

## 2 Materials and methods

### 2.1 Animals and experimental design

All experimental procedures were approved and conducted in accordance with the guidelines of the Animal Subjects Ethics Sub-Committee from The Hong Kong Polytechnic University and Hong Kong Science and Technology Park Corporation Institutional Animal Care and Use Committee. Wild-type mice (C57BL/6J) and retinal degeneration mice (rd10^*Pde*6*b*^) mice were bred in-house, and raised under a 12 h light/12 h dark cycle with *ad libitum* standard mouse chow and water. Animals were group-housed, in order to avoid the stress induced by social isolation.

### 2.2 Voluntary running protocol

**FIGURE 1:**
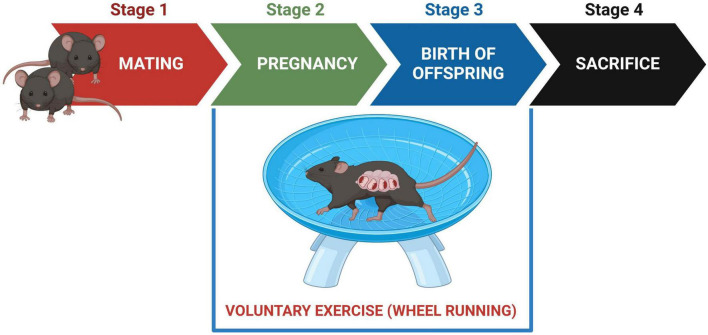
Experimental timeline. Stage 1: males were introduced to female breeders for mating. Stage 2: males were removed from the cages; All pregnant females had free access to running wheels for voluntary exercise; sedentary groups were given locked running wheels. Stage 3: running wheels remained in the cages until 6 weeks postnatal day. Stage 4: offspring were sacrificed at 6 weeks of age; Retinal tissues and serum samples were collected for analyses.

Following a 7 days acclimation period, adult rd10 males were introduced to adult rd10 female breeders. After confirming pregnancy by the presence of seminal plugs, adult male breeders were separated from the female breeders. Pregnant females were randomly assigned into four treatment groups [sedentary wild-type (*n* = 4), exercised wild-type (*n* = 4), sedentary rd10 (*n* = 4), exercised rd10 (*n* = 4)] and running wheels (Med-Associates, Inc., St. Albans, VT, United States) were introduced in each cage (Figure 1). Exercised groups had free access to running wheel while sedentary groups had locked running wheels in the holding cages. To confirm voluntary running activity, we recorded the daily running distances of each dam. The running wheels remained in the cages until the pups reached 6 weeks of age. Male and female offspring were euthanised at 6 weeks old by CO_2_ asphyxiation. The CO2 flow was maintained for at least 1–2 min after respiratory arrest to ensure complete euthanasia. Retinal and serum samples were collected for further analysis (Figure 1).

### 2.3 Tissue preparation for immunohistochemistry

Mice were deeply anesthetized with isoflurane (RWD Life Science, China) administered at 2%–3% for induction and 1%–2% for maintenance, using 100% medical oxygen as the carrier gas. Eyes for immunohistochemistry were quickly enucleated and fixed in 4% paraformaldehyde (PFA, sc-281692, Santa Cruz Biotechnology, United States) for 30 min at room temperature and washed with 1X phosphate buffer saline (PBS, pH 7.4, Gibco, Life Technologies, United States). After fixation, the eyes were transferred to 30% sucrose solution until they sank. In order to access the posterior hemispheres (containing the retinas), the anterior hemispheres (lens, iris-ciliary body, and cornea) were dissected. Subsequently, the posterior hemispheres were embedded in a cryomold containing a mixture of 30% sucrose and optimal cutting tissue solution (ratio 1:1). The retinas were sectioned (20 μm thickness) in a freezing (−*20*°C) cryostat (Leica, CM1950, Nussloch, Germany). Sections were obtained at comparable orientations to minimize variability due to sectioning angle and the sections were stored at −*80*°C until use.

### 2.4 Immunohistochemistry and confocal imaging

The retinal sections were washed and incubated at room temperature for 30 min with 0.1% Triton X-100/1X PBS to permeability the sections and ensure that the antibodies could access intracellular proteins. Non-specific interactions were blocked by covering retinal sections with 1X PBS containing 3% bovine serum albumin (BSA) and 0.1% Triton X-100. Retinal sections were incubated overnight (at 4°C) with primary antibodies to stain the rods’ outer segment (OS) (anti-rhodopsin mouse monoclonal antibody clone 4D2, 1:500, MABN15, Merck Millipore, Darmstadt, Germany) and cones’ OS (anti-cone arrestin rabbit polyclonal antibody, 1:500, AB15282, Merck Millipore, Darmstadt, Germany). Following overnight incubation, the tissues were washed in PBS and incubated with the corresponding secondary antibodies (Alexa Fluor^®^ 594-conjugated affinipure donkey anti-mouse IgG, 715-585-150 and Alexa Fluor^®^ 488-conjugated affinipure donkey anti-rabbit IgG, 711-545-152, Jackson ImmunoResearch, United States). Furthermore, the outer nuclear layer (ONL) was also stained with 4,6-diamidino-2-phenylindole (DAPI, D9542, 1:1000, Sigma-Aldrich, United States) to visualise the photoreceptors’ nuclei. After overnight incubation, the retinal sections were rinsed with PBS and cover slipped in aqueous fluorescent mounting media (HC08, Merck Millipore, Darmstadt, Germany).

Confocal imaging of immunofluorescent positive staining from the retinal sections were captured using Zeiss LSM 800 Airyscan Confocal Microscope (Carl Zeiss, Oberkochen, Germany). To ensure consistency in photoreceptor nuclei quantification and retinal cross-sections, a Plan Apochromat 20X/0.8 NA objective was used to capture 200 μm of three different regions of the retinas: Central retina (at 50 μm from the optic nerve head), left peripheral end and right peripheral end. These regions were used to obtain the mean photoreceptor number. In order to assess the survival of the photoreceptors (rods and cones), the number of the photoreceptor nuclei in the ONL was counted. Furthermore, starting from one end of each 200 μm region, the length of the photoreceptors’ OS was measured at intervals of 50 μm and averaged to obtain the mean length ImageJ software (NIH, Bethesda, MA). Was used for all measurements and double-blind counting system was employed for all analyses. Although we did not normalize photoreceptor nuclei counts to the total inner retinal area (nerve fibre layer to the outer plexiform layer), we ensured that measurements were taken from equivalent retinal regions across all samples. This approach minimizes variability while preserving the ability to detect differences between experimental groups. Future studies may consider normalizing nuclei counts to inner retinal area to further account for potential sectioning inconsistencies.

### 2.5 Tissue preparation for protein analysis

Fresh retinal tissues were dissected from enucleated eyes and the total proteins were extracted from the freshly isolated retinas. The tissues were lysed for 40 min using a chilled radioimmunoprecipitation assay (RIPA) buffer (Santa Cruz Biotechnology, United States). This buffer was supplemented with protease and phosphatase inhibitor cocktails, as well as phenylmethanesulfonyl fluoride (Santa Cruz Biotechnology, United States). During the lysis process, the tissues were vortexed every 5 min. Samples were homogenized in 2 ml tubes [at 5,800 rpm, 4°C for 30 s and two cycles (frozen time per cycle: 20 s)] using Precellys^®^ Evolution Homogenizer (Bertin, Montigny-le-Bretonneux, France) and cleared by centrifugation (21.380 × *g*) at 4°C for 30 min. The supernatant protein was collected and stored at −*80*°C. The protein concentration was measured using the BCA Protein Assay Kit (Merck Millipore, Darmstadt, Germany). Blood samples were collected by cardiac puncture with a 25-G needle and 1 mL syringe. The samples were allowed to clot for 30 min at room temperature. Blood sera were collected by centrifugation at 3,000 rpm and 4°C for 15 min and stored at −*80*°C until needed.

### 2.6 Enzyme-linked immunosorbent assay (ELISA) measurement

Adiponectin levels in the serum/retinas were quantified by using the Adiponectin (mouse) ELISA Kit (Adipogen^®^, Life Sciences, Füllinsdorf, Switzerland). Adiponectin levels were determined by sandwich ELISA method according to kit manufacturer’s instructions.

### 2.7 Western blot analysis

Prior to electrophoresis, retinal supernatant samples were heat-denatured at 95°C for 5 min in Laemmli sample buffer containing 2% SDS and 5% β-mercaptoethanol to ensure complete protein denaturation and reduction of disulfide bonds. A total of 30 μg of total protein from each retinal sample were loaded on a 12% SDS-PAGE gel and transferred onto polyvinylidine fluoride membranes (BioRad, Hercules, California, United States). After 1 h of blocking with 5% non-fat dry milk, the membranes were washed in 0.1% PBS-Tween and incubated overnight at 4°C with primary antibodies for PGC-1α (rabbit polyclonal antibody, 1:500, A12848, MA, United States), AMPK (AMPK-α 1/2 mouse monoclonal antibody [D-6], 1:500, sc-525713, Santa Cruz Biotechnology, United States) and GAPDH (mouse monoclonal antibody, 1:500, sc-47724, Santa Cruz Biotechnology, United States). The next day, the membranes were incubated for 1 h with the corresponding HRP-labelled secondary antibodies (1:1000). The peroxidase reaction was visualized with an ECL kit (Ultrasense Pico Western Substrate, ECL01-50, Bioland Scientific, United States). Densiometric analysis was performed using ImageJ software (NIH, Bethesda, MA).

### 2.8 Statistical analyses

Statistical analyses to determine significant differences in the data means from the four groups were performed using Origin (OriginLab). Data are presented as means ± SEMs. Appropriate ANOVAs with *post-hoc* tests were performed for multiple comparisons. Non-parametric data were statistically analyzed with Mann-Whitney U test. Any *p*-values < 0.05 were considered as significant.

## 3 Results

### 3.1 Voluntary exercise preserves photoreceptor number in rd10 retina

**FIGURE 2:**
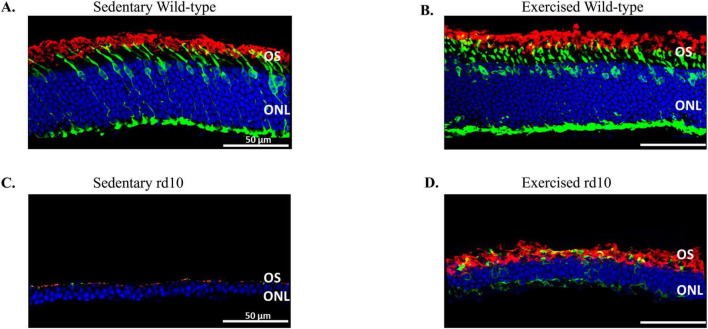
Representative immunofluorescent images of retinal cross-sections co-stained with rhodopsin (red), mouse cone arrestin (green) and DAPI (blue). **(A)** Sedentary wild-type mouse, **(B)** Exercised wild-type mouse, **(C)** Sedentary rd10 mouse, and **(D)** Exercised rd10 mouse. Voluntary exercise rescued loss of outer nuclei layer (ONL), rods and cones outer segment (OS) when compared to sedentary rd10 mice.

**FIGURE 3:**
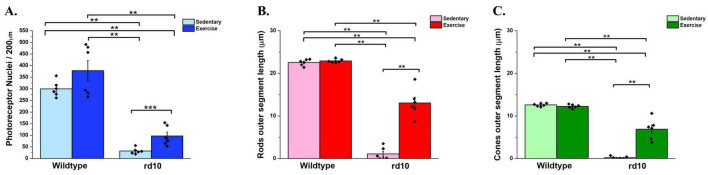
Photoreceptors’ nuclei and outer segment quantification. **(A)** Density of photoreceptor nuclei; **(B)** Rods’ outer segment length and **(C)** Cones’ outer segment length. Voluntary exercise did not show significant effects on the density of photoreceptor nuclei, rods’ outer segment length and cones’ outer segment length in wild-type mice. However, voluntary exercise showed significant effect in preserving photoreceptor nuclei (*p* = 0.004), rods’ outer segment (*p* = 0.002) and cones’ outer segment length (*p* = 0.002) in rd10 mice when compared to sedentary rd10 mice, though there were still significant higher number of photoreceptor nuclei, longer rods’ outer segment and cones’ outer segment length in wild-type mice. *n* = 6 mice offspring from four mice dams per group. Asterisk indicates significant difference ** *p* < 0.05, *** *p* < 0.005.

We investigated the neuroprotective effects of voluntary wheel running exercise on photoreceptor survival in young adult rd10 mouse model of RP. Pregnant females were randomly assigned into four treatment groups and were allowed to voluntarily run until their pups were reached 6 weeks of age. Analysis of running wheel data confirmed that exercised dams engaged in voluntary running throughout the study. Exercised rd10 dams exhibited the highest average running distance (11.1 km/day), while exercised wild-type dams ran an average of 4.7 km/day. Individual variability was observed, with running distances ranging from 6.8 to 16 km/day in rd10 dams and 0.2 to 7.2 km/day in wild-type dams (Table 1). Sedentary dams (both wild-type and rd10) did not engage in any running activity (Table 1). These data confirm that maternal exercise occurred consistently, supporting our hypothesis that prenatal exercise influences offspring retinal health. At 6 weeks of age, the offspring were sacrificed and their retinas were subjected to immunohistochemistry. Wild-type mice did not exhibit a significant difference in photoreceptor nuclei counts (Figures 2A, B). Although photoreceptor loss was apparent in exercised or sedentary rd10 mice, exercised rd10 mice had higher density of ONLs when compared to sedentary rd10 (Figures 2C, D). A Kruskal-Wallis test was conducted to compare the distributions of photoreceptor nuclei counts across the four groups. The test revealed a statistically significant difference between the groups, χ^2^(3) = 19.45, *p* < 0.001. Further analysis revealed that exercised rd10 mice had significant greater number of photoreceptor nuclei (97 ± 16) compared to sedentary rd10 mice (32 ± 5) (Figure 3A; Mann Whitney U-test U = 35, Z = 2.64, *p* = 0.004). The analysis of the lengths of cones and rods in the OS revealed that exercised rd10 mice displayed significantly longer rods (13.1 ± 1.2 μm) (Figure 3B; Kruskal-Wallis test χ^2^(3) = 19.48, *p* < 0.001; Mann Whitney U-test U = 36, Z = 2.81, *p* = 0.002) and cones (7 ± 0.9 μm) (Figure 3C; Kruskal-Wallis test χ^2^(3) = 19.9, *p* < 0.001; Mann Whitney U-test U = 36, Z = 2.81, *p* = 0.002).

**TABLE 1:** Summary of daily running distances in wild-type and rd10 mice under sedentary and exercise conditions.

Mouse group	Dam 1 (kilometres/day)	Dam 2 (kilometres/day)	Dam 3 (kilometres/day)	Dam 4 (kilometres/day)	Average distance per day (kilometres/dam)
Sedentary wild-type	0	0	0	0	0
Exercised wild-type	0.2	5.8	7.2	5.6	4.7
Sedentary rd10	0	0	0	0	0
Exercised rd10	13.8	16	7.7	6.8	11.1

Values represent the distance (in kilometers) run per day by individual dams across different experimental groups. The average distance per day was calculated for each group. Wild-type and rd10 mice were either maintained under sedentary conditions (0 km/day) or subjected to voluntary running.

### 3.2 Voluntary exercise elevates serum adiponectin levels in rd10 mice

**FIGURE 4:**
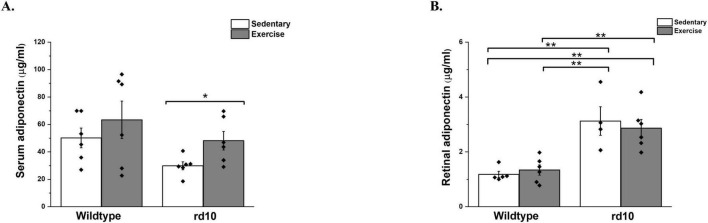
Changes in serum adiponectin levels of the wild-type and rd10 mice. **(A)** Serum adiponectin levels increased significantly in exercised rd10 mice compared to sedentary rd10 mice (*p* = 0.04), with a significant increase in **(B)** Retinal adiponectin levels when compared with exercised wild-type mice (*p* < 0.005). Rd10 mice showed significantly higher levels of retinal adiponectin when compared to the wild-type mice (*p* < 0.005). Voluntary exercise significantly increased serum adiponectin levels in rd10 exercise group compared to their sedentary counterparts, but showed no effect in the wild-type mice. Asterisk indicates significant difference * *p* < 0.04, *** *p* < 0.005.

Next, we tested whether voluntary exercise induces retinal neuroprotection via increasing levels of adiponectin. Results showed significant main effects of exercise on adiponectin levels in the serum [Welch’s ANOVA, F(3, 11) = 5.88, *p* = 0.012] and in the retina [two-way ANOVA, F(1, 20) = 7.59, *p* = 0.013] of rd10 mice (Figures 4A, B, respectively). In exercised rd10 mice, voluntary exercise significantly increased serum adiponectin levels compared to sedentary rd10 (Figure 4A; Mann Whitney U-test U = 31, Z = 2, *p* = 0.04). Increase in retinal adiponectin levels was observed in both sedentary and exercised rd10 mice compared with sedentary and exercised wild-type mice (Figure 4B; Mann Whitney, *p* < 0.005 in all pairwise comparisons). Exercise did not show significant effect on altering serum and retinal adiponectin levels in wild-type C57 mice (Figures 4A, B).

### 3.3 Voluntary exercise shows no effect on retinal AMPK and PGC-1α of rd10

**FIGURE 5:**
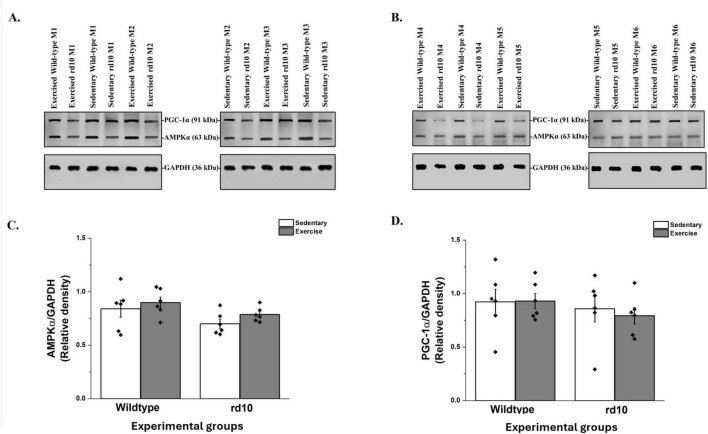
Western blotting analysis of retinal peroxisome proliferator-activated receptor-gamma coactivator-1-alpha (PGC-1α) and AMP-activated protein kinase (AMPK) in wild-type and rd10 mice. **(A)** PGC-1α and AMPK protein bands from experimental mice M1–M3; **(B)** PGC-1α and AMPK protein bands from experimental mice M4–M6. GAPDH served as a loading control for the gels. M indicates mouse; *n* = 6 mice offspring from four DAMS per group; **(C)** Relative level of retinal AMPK protein in experimental mice groups; **(D)** Relative level of retinal PGC-1α protein in experimental mice groups. Data shown as a scatter plot with mean ± SEM. No significant difference in retinal AMPK (*P* = 0.794) and PGC-1α (*P* = 0.724) was observed across all groups of wild-type and rd10 mice. Voluntary exercise did not significantly alter the levels of PGC-1α and AMPK in the retina of wild-type and rd10 mice; *n* = 6 mice offspring from four dams per group.

The AMPK/PGC-1α signaling pathway is involved in adiponectin receptor-mediated signaling transduction (Jäger et al., 2007; Balasubramanian et al., 2022). Results showed that voluntary exercise did not significantly affect retinal protein expression of AMPK and PGC-1α in rd10 mice (Figures 5A, B). Moreover, there was no change in retinal AMPK [two-way ANOVA, F(1, 20) = 0.07, *p* = 0.794] and PGC-1α [two-way ANOVA; F(1, 20) = 0.13, *p* = 0.724] levels, respectively (Figures 5C, D).

## 4 Discussion

The present study provides evidence for the neuroprotective effects of voluntary exercise on photoreceptor survival in a transgenic mouse model of RP with severe rod and cone cell death. Our findings demonstrated exercise-induced retinal neuroprotection, supporting the potential effectiveness of a physical exercise intervention to protect against retinal cell death associated with RP.

Our morphological analysis revealed significant preservation of photoreceptor nuclei in exercised rd10 mice compared to their sedentary counterparts. This preservation was evident in the quantitative count of photoreceptor nuclei in the ONL. Moreover, the exercised rd10 mice exhibited significantly longer rod and cone OS. Our measurement of OS length was based on histological sections, which, while commonly used in retinal studies, may be affected by expansion and contraction during tissue processing. This could introduce variability in absolute measurements. Optical coherence tomography (OCT) would provide *in vivo* measurements that avoid these artifacts and allow for longitudinal assessments of retinal structure. Future studies should consider incorporating OCT imaging prior to histological analysis to further validate exercise-induced preservation of photoreceptor morphology.

Our results are consistent with previous studies that have demonstrated the neuroprotective effects of exercise in retinal degeneration models. Our findings demonstrate that maternal voluntary exercise confers neuroprotective effects on photoreceptor survival in rd10 offspring. These results expand upon previous studies investigating the effects of postnatal voluntary exercise in RP models. Notably, Hanif et al. (2015) reported similar photoreceptor preservation in rd10 mice when pups were provided with running wheels post-weaning. However, our study differs in that we specifically examined the effects of maternal exercise during pregnancy, rather than postnatal exercise by the offspring. This distinction is important, as maternal exercise has been shown to induce systemic metabolic changes, including increased circulating neurotrophic factors and adiponectin, which may influence foetal development and retinal resilience (Yau et al., 2019). This transgenerational effect of exercise on retinal health opens up new avenues for potential preventive strategies in RP management. While our study focused on the rd10 model, similar neuroprotective effects of exercise have been observed in other retinal degeneration models. For example, a recent study demonstrated that voluntary exercise preserves visual function and reduces inflammatory response in an adult mouse model of autosomal dominant RP (Bales et al., 2024). Additionally, aerobic exercise has been shown to protect retinal function and structure in a light-induced retinal degeneration mouse model, by preserving photoreceptor cell counts and retinal layers (Lawson et al., 2014). This consistency across different models strengthens the potential therapeutic effects of exercise interventions in various forms of retinal degeneration.

Our running wheel data confirmed that rd10 dams engaged in more voluntary running than WT dams, with an average daily distance of 11.1 km/day compared to 4.7 km/day in WT dams. This increased activity may reflect behavioral differences in rd10 mice, possibly linked to compensatory mechanisms associated with visual impairment (Strettoi et al., 2002; Phillips et al., 2010). The observed photoreceptor preservation in offspring of exercised dams suggests that maternal exercise may exert protective effects through circulating factors, independent of post-weaning voluntary activity. However, it is also possible that postnatal voluntary activity contributed to the observed neuroprotection, as previous studies have reported that post-weaning exercise can enhance retinal resilience in RP models. While our study primarily focused on maternal exercise, we cannot rule out the potential additive effects of postnatal activity in rescuing both rod and cone degeneration. Exercise is known to promote mitochondrial biogenesis, enhance neurotrophic signaling, and reduce oxidative stress, all of which could play a role in preserving photoreceptor integrity. Clinically, these findings have potential implications for individuals with RP who are considering pregnancy. As RP is an inherited disorder, maternal lifestyle interventions such as regular physical activity during pregnancy may offer a non-invasive strategy to enhance retinal resilience in offspring. While additional studies are needed to determine the exact mechanisms and translational potential of maternal exercise in human RP, our study provides novel insights into its possible role as a protective intervention. Future research should aim to delineate the relative contributions of prenatal and postnatal exercise to retinal neuroprotection. Longitudinal studies incorporating both maternal and post-weaning exercise paradigms, as well as assessments of circulating metabolic and neurotrophic factors, could provide a more comprehensive understanding of how physical activity influences RP progression. In particular, future studies should investigate whether postnatal voluntary running initiated at 4 weeks of age, when pups are physically capable of engaging in exercise, can mitigate the severe rod and cone degeneration observed at earlier time points. This approach would help delineate the specific contributions of postnatal exercise in comparison to maternal exercise. Additionally, controlling for stress-related factors introduced by premature wheel removal will be important to ensure that observed effects are attributed to exercise rather than environmental stressors.

Our study found a significant elevation of adiponectin levels in both serum and retina of exercised rd10 mice, but not wild-type mice, indicating a specific response in the RP model. The exercise-induced elevation of serum adiponectin levels aligns with previous studies demonstrating its neuroprotective properties in various neurological disorders. For example, a systematic review and meta-analysis confirmed that physical exercise, particularly aerobic exercise, significantly increases adiponectin levels in prediabetic and diabetic adults (Becic et al., 2018; Otu and Otu, 2021). However, retinal adiponectin levels were elevated in both sedentary and exercised rd10 mice relative to wild-type controls, suggesting that retinal adiponectin accumulation is more likely a pathological response rather than an effect of exercise. The lack of increased adiponectin in the exercised WT group was not unexpected, as previous studies have shown that baseline adiponectin levels in healthy animals remain relatively stable and may not respond to exercise unless metabolic stressors are present (Polak et al., 2006). In contrast, in degenerative disease models like rd10, systemic metabolic alterations may increase adiponectin responsiveness to exercise. This increase in adiponectin is associated with its neuroprotective properties, as adiponectin plays a crucial role in the central nervous system by influencing synaptic plasticity and energy homeostasis (Yau et al., 2014; Bloemer et al., 2018; Formolo et al., 2022). Moreover, in Alzheimer’s disease and Parkinson’s disease models, adiponectin crosses the blood-brain barrier to reduce inflammation and oxidative stress (Ng and Chan, 2017; Polito et al., 2020; Formolo et al., 2022). Consequently, adiponectin may act as a key mediator in exercise-induced retinal neuroprotection.

Interestingly, our study also observed elevated retinal adiponectin levels in both sedentary and exercised rd10 mice compared to wild-type controls. This suggests that the increase in retinal adiponectin is inherent to the RP pathology rather than being induced by voluntary exercise. It is plausible that this elevation represents a compensatory mechanism associated with retinal degeneration. Despite the absence of increased retinal adiponectin expression in exercised rd10 mice, adiponectin’s neuroprotective role may still be plausible through indirect mechanisms. Circulating adiponectin is known to modulate inflammation and oxidative stress via activation of its receptors, AdipoR1 and AdipoR2, which are expressed in the retina (Rice et al., 2015). Given that previous research has demonstrated adiponectin-mediated neuroprotection in retinal degeneration models (Fu et al., 2016), it is possible that systemic adiponectin exerts protective effects through pathways independent of its direct increase in retinal tissue. Further studies are needed to evaluate whether adiponectin receptor activation and downstream signaling play a role in mediating exercise-induced neuroprotection.

The role of adiponectin in retinal health has been previously explored in other contexts. For instance, it has been demonstrated that adiponectin mediates the protective effects of dietary omega-3 long-chain polyunsaturated fatty acid against choroidal neovascularization in mice (Fu et al., 2017). These findings highlight the multifaceted role of adiponectin in ocular diseases. Our present findings provide novel insights into the molecular pathways involved in exercise-mediated benefits in RP. By linking exercise-induced serum adiponectin elevation to photoreceptor preservation, this study emphasizes the potential systemic benefits of physical activity for retinal health. However, while our findings establish a strong correlation between increased circulating adiponectin and photoreceptor preservation, they do not conclusively demonstrate that adiponectin is the primary driver of the observed neuroprotection. Exercise induces a broad range of systemic changes, including elevated BDNF, IGF-1, and anti-inflammatory cytokines, which may also contribute to retinal resilience. Future studies using adiponectin-deficient models or pharmacological inhibition of adiponectin signaling will be necessary to determine whether adiponectin plays a direct causative role in exercise-induced neuroprotection or functions as part of a broader network of exercise-induced metabolic adaptations.

The observation that retinal AMPK and PGC-1α levels did not significantly change in response to exercise in our rd10 mouse model of RP warrants further discussion, especially in light of the significant photoreceptor preservation and increased adiponectin levels we observed. Several factors could contribute to this seemingly contradictory result. One crucial consideration is the cellular composition of the retina and the limitations of whole-tissue analysis. Photoreceptors, while critical for vision, comprise only a fraction of the total retinal cell population. In mice, photoreceptors make up approximately 70% of retinal cells, with rods being the predominant type (Dieterich et al., 2002; Masland, 2012). However, in the rd10 model, where photoreceptor degeneration is a hallmark, this percentage is lower. Consequently, when analyzing whole retinal lysates via Western blotting in our study, changes specific to photoreceptors might be diluted or masked by the protein content of other retinal cell types. This “dilution effect” has been recognized as a limitation in retinal research. For instance, it has been noted that in studying cone survival in RP models, the use of whole retinal extracts could obscure cone-specific changes due to the overwhelming presence of rod-derived proteins (Cronin et al., 2010). One limitation of our study is that whole-retinal lysates may introduce a dilution effect, potentially masking photoreceptor-specific changes in AMPK and PGC-1α expression. Given that retinal tissue comprises multiple cell types, including Müller glia and vascular endothelial cells, subtle molecular changes within the photoreceptor layer may be less detectable in homogenized samples. Future studies should incorporate immunohistochemical analyses or laser capture microdissection to better resolve cell-specific expression patterns. Nonetheless, our findings align with previous reports suggesting that AMPK and PGC-1α activation in the retina may be influenced by systemic metabolic regulators rather than being confined to photoreceptors alone (Herzig and Shaw, 2018). To address this issue, future studies could employ more targeted approaches. Laser capture microdissection, for example, allows for the isolation of specific retinal layers or cell types before protein analysis, potentially revealing photoreceptor-specific changes in AMPK and PGC-1α that were not detectable in our whole-retina analysis.

Another factor to consider is the distribution of adiponectin receptors within the retina. While our study demonstrated increased adiponectin levels in exercised rd10 mice, the expression pattern of AdipoR1 and AdipoR2 across different retinal cell types could influence the observed effects. Recent research has shed light on the distribution of these receptors in the brain and retina. For instance, it has been reported that both AdipoR1 and AdipoR2 are widely distributed in adult mouse brains, with expression primarily in neurons and blood vessels (Clain et al., 2022). In the retina, adiponectin receptors, particularly AdipoR1, are expressed in photoreceptors and RPE cells. Studies have shown that AdipoR1 is predominantly localized at the interface between the RPE apical processes and the distal part of the photoreceptor outer segments, with significantly higher expression in RPE cells compared to the neural retina (Lewandowski et al., 2022). This localization suggests a critical role for AdipoR1 in maintaining photoreceptor function and retinal health. AdipoR2 is also expressed in the retina, but is primarily localized in the RPE. The differential expression of these receptors may result in varying adiponectin-mediated effects across different retinal cell types. If AdipoR1 and AdipoR2 expression is relatively low in photoreceptors compared to other retinal neurons, the adiponectin-mediated activation of pathways such as AMPK and PGC-1α could be more pronounced in non-photoreceptor cells (Ruiz et al., 2019; Miyagishima et al., 2021). This could explain why significant changes might not be observed in whole-retina analyses but could be present at a cellular level. Moreover, the temporal dynamics of AMPK and PGC-1α activation in response to exercise and increased adiponectin levels should be considered (Chen et al., 2022; Shukal et al., 2022). It’s possible that these proteins undergo transient changes that were not captured at our chosen time point for analysis. It has been demonstrated in skeletal muscle that exercise-induced changes in AMPK and PGC-1α can be rapid and transient (Brandt et al., 2017; Gurd et al., 2023). Similar dynamics might occur in the retina, necessitating a time-course study to fully capture the molecular responses to exercise. Additionally, the complex pathophysiology of RP in the rd10 model might influence how retinal cells respond to exercise-induced adiponectin increases (Egger et al., 2012; Zhou et al., 2024). The ongoing stress and degeneration in photoreceptors could alter their responsiveness to adiponectin signaling or activate compensatory mechanisms that maintain baseline levels of AMPK and PGC-1α despite increased adiponectin (Kaarniranta et al., 2018).

Given the significant loss of photoreceptors by 6 weeks (postnatal day P42) in rd10 mice, earlier time points such as 3 weeks (P21) could provide additional insights into the progression of neuroprotection. Assessing an earlier stage could help determine whether exercise-induced benefits emerge before substantial degeneration occurs and reduce variability associated with advanced cell loss. However, our study focused on P42 to evaluate the long-term effects of maternal exercise on photoreceptor survival. Future studies should consider a time-course analysis to better understand the temporal dynamics of exercise-induced neuroprotection in RP.

## 5 Conclusion

This study on exercise-induced neuroprotection in RP highlights the beneficial effects of voluntary exercise on photoreceptor preservation in the rd10 mouse model. The findings demonstrate that exercise significantly increases photoreceptor nuclei density and outer segment lengths in these mice, suggesting a protective role against retinal degeneration. Notably, serum adiponectin levels were elevated in exercised rd10 mice, but retinal adiponectin levels remained unchanged following exercise, suggesting that systemic rather than local adiponectin signaling may contribute to the observed neuroprotection. Since no direct evidence of adiponectin receptor activation was measured in this study, future investigations are necessary to determine whether maternal exercise-induced neuroprotection in RP is mediated through adiponectin-dependent pathways. Importantly, the elevated retinal adiponectin levels in both sedentary and exercised rd10 mice suggest that this increase is inherent to the RP pathology and not solely exercise-induced. Despite these promising outcomes, no significant changes were observed in the expression of AMPK and PGC-1α, key regulators of mitochondrial biogenesis pathways, suggesting that the neuroprotective effects may occur through alternative mechanisms. Moreover, total protein levels alone may not fully capture the activation status of these pathways. AMPK is activated via phosphorylation at Thr172, and PGC-1α function is regulated through phosphorylation and deacetylation. Future studies should assess phosphorylated AMPK (p-AMPK/AMPK ratio) and phosphorylated PGC-1α, as well as downstream targets such as NRF1, TFAM, and mitochondrial respiratory chain genes, to determine whether maternal exercise influences mitochondrial biogenesis at a post-translational level. Investigating these regulatory mechanisms would provide deeper insights into the metabolic effects of exercise in RP models.

While our findings demonstrate that voluntary exercise preserves photoreceptors in rd10 offspring, the underlying molecular mechanisms remain to be fully elucidated. Apoptosis is a key driver of photoreceptor degeneration in rd10 mice, and future studies should investigate apoptotic markers such as cleaved caspase-3 and the Bax/Bcl-2 ratio to determine whether voluntary maternal exercise confers neuroprotection through the modulation of apoptotic pathways. Incorporating these molecular analyses would provide further mechanistic insight into the anti-apoptotic effects of maternal exercise in retinal degeneration. These results emphasize the potential of exercise as a therapeutic strategy for RP and highlight adiponectin as a promising target for future research aimed at elucidating the molecular pathways underlying retinal neuroprotection. Although our study focused on adiponectin signaling, it is possible that additional pathways contribute to the neuroprotective effects of maternal exercise. Exercise has been shown to upregulate neurotrophic factors such as BDNF and CNTF (Supplementary Figure 1), which play critical roles in neuronal survival and photoreceptor preservation. BDNF has been implicated in retinal neuroprotection, whereas CNTF has been shown to support retinal ganglion cell survival. Additionally, exercise is known to exert anti-inflammatory effects, which may help mitigate retinal degeneration. Future studies should evaluate whether these neurotrophic and inflammatory pathways contribute to the observed photoreceptor preservation to gain a more comprehensive understanding of the mechanisms underlying exercise-induced neuroprotection in RP.

## Data Availability

The original contributions presented in this study are included in this article/Supplementary material, further inquiries can be directed to the corresponding author.
