# Supplementary figures and images for: Voluntary running partially prevents photoreceptor cell death in retinitis pigmentosa
Source: Front Neurosci. 2025 Apr 25;19:1563607. doi: 10.3389/fnins.2025.1563607 (PMC12062024; doi:10.3389/fnins.2025.1563607)

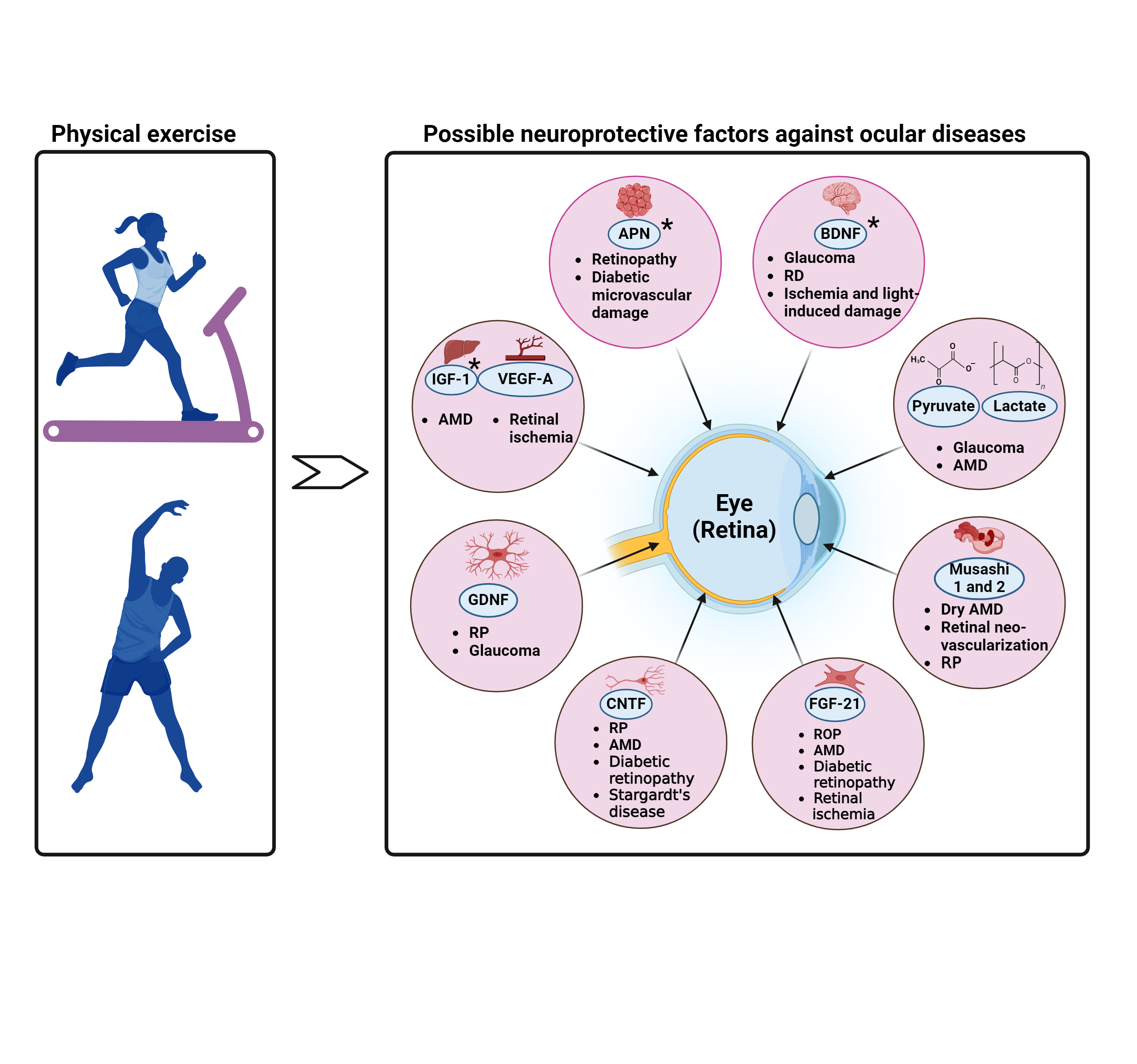

Supplement: Supplementary file 1 [file Image_1.tif]
